# Altered Resting-State Functional Connectivity of Multiple Networks and Disrupted Correlation With Executive Function in Major Depressive Disorder

**DOI:** 10.3389/fneur.2020.00272

**Published:** 2020-04-28

**Authors:** Yujie Liu, Yaoping Chen, Xinyu Liang, Danian Li, Yanting Zheng, Hanyue Zhang, Ying Cui, Jingxian Chen, Jiarui Liu, Shijun Qiu

**Affiliations:** ^1^First Clinical Medical College, Guangzhou University of Chinese Medicine, Guangzhou, China; ^2^Department of Radiology, The First Affiliated Hospital of Guangzhou University of Chinese Medicine, Guangzhou, China; ^3^Cerebropathy Center, The First Affiliated Hospital of Guangzhou University of Chinese Medicine, Guangzhou, China; ^4^Cerebropathy Center, The Third Affiliated Hospital of Guangzhou Medical University, Guangzhou, China; ^5^Department of Radiology, Shunde Hospital of Southern Medical University, Shunde, China; ^6^Department of Radiology, Zhuhai Hospital of Southern Medical University, Zhuhai, China

**Keywords:** major depressive disorder, functional connectivity, resting state, fMRI, neuropsychological test, executive function

## Abstract

**Background:** Major depressive disorder (MDD) is one of the most common and costly psychiatric disorders. In addition to significant changes in mood, MDD patients face an increased risk of developing cognitive dysfunction. It is important to gain an improved understanding of cognitive impairments and find a biomarker for cognitive impairment diagnosis in MDD.

**Methods:** One hundred MDD patients and 100 normal controls (NCs) completed resting-state fMRI (rs-fMRI) scan, in which 34 MDD patients and 34 NCs had scores in multiple cognitive domains (executive function, verbal fluency, and processing speed). Twenty-seven regions of interest from the default mode network (DMN), central executive network (CEN), salience network (SN), and limbic system (LS) were selected as seeds for functional connectivity (FC) analyses with the voxels in the whole brain. Finally, partial correlations were conducted for cognitive domain scores and FCs with significant differences between the MDD and NC groups.

**Results:** Significant FC differences between groups were identified among the seeds and clusters in the DMN, CEN, LS, visual network, somatomotor network, ventral attention network, and dorsal attention network. In the MDD patients, the magnitude of the Stroop interference effect was positively correlated with the illness duration, and the illness duration was negatively correlated with the FC between the right ventral hippocampal gyrus and the left inferior frontal gyrus. However, the correlation between the Stroop interference effect and the FC of the right anterior prefrontal cortex with the left cerebellum_4_5 was disrupted in these patients.

**Conclusions:** The MDD patients have altered FCs among multiple brain networks and a disrupted correlation between the FC of prefrontal cortex and executive function. The disrupted correlation could present before the symptoms develop and may be the core process in the development of executive function impairment.

## Introduction

Major depressive disorder (MDD) is one of the most common and costly psychiatric disorders (1). This condition has been ranked as one of the top 10 leading causes of disability among 191 countries (2) and is the second leading cause of disability worldwide, affecting 4.7% of the global population (3). MDD patients suffer significant changes in mood, characterized by sadness along with various other symptoms, such as fatigue, altered appetite, and/or sleep (4). Moreover, cognitive impairments are commonly detected in MDD patients (5–7). In a recent study, cognitive impairments related to MDD were grouped into four cognitive domains: (i) verbal learning and memory, (ii) visuospatial learning and memory, (iii) executive function (EF)/attention, and (iv) psychomotor speed; 8.9–37.5% of MDD patients were impaired in two or more cognitive domains (8). High rates of persisting cognitive impairments were also found in MDD patients that nearly 60% of MDD patients remained cognitively impaired at 6-month follow-up (9). There are data suggesting that proper medical treatment may lead to improved cognitive functioning (10–12), while worse treatment outcomes and higher rates of recurrence are associated with poorer cognition (13). In light of these findings, it is important to gain an improved understanding of cognitive impairments in MDD and find a potential biomarker for cognitive impairment diagnosis in MDD, which could be of great clinical importance in terms of allowing early, accurate diagnosis and reducing the chances of chronic relapse and recurrence (5).

Resting-state functional MRI (rs-fMRI) has been widely used to investigate the neural mechanisms of brain dysfunctions (14) and to explore potential imaging biomarkers in various diseases (e.g., MDD, social anxiety disorder, and Alzheimer's disease) (15–17). By measuring fluctuations in blood-oxygen-level-dependent (BOLD) signals, rs-fMRI can be used to assess brain functional connectivity (FC). Researchers have indicated that cognitive impairments in MDD are related to significant FC changes within and between several brain networks, such as the default mode network (DMN), central executive network (CEN), salience network (SN), and limbic system (LS) (18–23). For example, the fronto-limbic system, a key network for emotional regulation and memory, is potentially linked to cognitive impairment in unmedicated MDD patients (24). The alterations in the large-scale brain FC network in MDD may demonstrate the depressive biases toward internal thoughts at the cost of engaging with the external world, resulting in lapses in cognitive problems (15, 25).

However, limitations exist in the previous studies. First, most studies that used seed-based methods to examine the whole-brain voxel-wise FC alteration in MDD only chose seeds from a single network, but seeds from multiple cognition-related networks would be more helpful in detecting the cognitive impairments. Second, the sample size in the previous studies is relatively small, and the majority of the patients in the study have a treatment history. In this scenario, the medication effect cannot be distinguished from those associated with the disease itself. Therefore, a larger sample composed purely of first-episode and drug-naïve MDD patients may eliminate possible confounding factors such as different episodes and medication use and achieve a more reliable result. In our previous study (26), we applied spectral dynamic causal modeling to estimate the effective connectivity of a large-scale network consisting of 27 ROIs (from the DMN, CEN, SN, and LS) in the 100 MDD patients and 100 NCs, and found that reduced excitatory and increased inhibitory connections coexisted within the DMN, underlying disrupted self-recognition and emotional control in MDD. We also proposed a new dynamic FC-based metric, high-order FC (27), to measure the temporal synchronization of long-range FC dynamics; we found that high-order FC significantly improved MDD diagnostic accuracy compared to conventional FC (28). Since the above studies did not analyze cognitive performance in MDD patients, we will do so in this paper.

In this study, we hypothesized that the FC alterations of four likely involved resting-state networks (DMN, CEN, SN, and LS) were associated with the cognitive impairments in first-episode and drug-naïve MDD patients. We applied a seed-based method to examine the whole-brain voxel-wise FC of 27 predefined seeds from DMN, CEN, SN, and LS based on rs-fMRI data collected from a relatively large sample size, including 100 first-episode and drug-naïve MDD and 100 normal controls (NCs). We also correlated the significantly altered FC with scores on a battery of neuropsychological tests that covered cognitive domains including executive function, verbal fluency, and processing speed in 34 MDD patients and 34 NCs. The results showed that MDD patients possessed altered FCs in multiple brain networks and a disrupted correlation between the FC of prefrontal cortex and executive function. The disrupted correlation could present before the symptoms develop and may be the core process in the development of executive function impairment.

## Methods and Materials

### Participants

A total of 119 first-episode, treatment-naïve MDD patients and 109 NCs from two datasets were used in this study. MDD patients were recruited from the psychological counseling outpatient clinic of the First Affiliated Hospital of Guangzhou University of Chinese Medicine from August 2015 to June 2018. The diagnosis of treatment-naïve, first-episode depression was made by two attending psychiatrists, each of whom had more than 10 years of experience with the Diagnostic and Statistical Manual of Mental Disorders (DSM)-5 (29); the Structured Clinical Interview for the DSM (SCID) was used to assess whether the diagnostic criteria were met (30). The 17-item Hamilton Depression Rating Scale (HDRS-17) (31) was also used to evaluate the severity of depression (32). Each patient self-reported a rough estimate of illness duration. The other inclusion criteria for MDD patients were as follows: (1) aged between 18 and 55 years old, (2) HDRS-17 score > 17, (3) right-handed native Chinese speaker, and (4) free of any history of neurological illness or any other psychiatric disorder according to the DSM-5. Exclusion criteria included (1) a history of any significant illness, (2) alcohol abuse [a total score ≥ 8 on the Alcohol Use Disorders Identification Test (33)], and (3) contraindications to MRI scans. The NCs were all volunteers who were physically healthy based on their self-reported medical history and mentally healthy according to the Mini-International Neuropsychiatric Interview (MINI) (34) as applied by two psychologists. Besides, the HDRS-17 score of NCs was <7. This study was conducted in accordance with the Declaration of Helsinki. All participants provided written informed consent, and the study was approved by the Ethics Committee of the First Affiliated Hospital of Guangzhou University of Chinese Medicine, Guangzhou, China.

### Clinical Assessment and Neuropsychological Testing

Participants in the second dataset (37 MDD patients and 37 NCs) were assessed by a battery of neuropsychological tests that covered cognitive domains including executive function, verbal fluency, and processing speed. The tests were administered by a trained psychometric technician supervised by a clinical neuropsychologist.

First, participants were subjected to a Stroop Color-Word Test (SCWT) (35), as research has consistently found inhibitory control impaired in MDD (36), and we hypothesized that the FC changes were associated with this cognitive impairment. The SCWT included three parts. Two of them represent the “congruous condition” in which participants were required to read out the name of a color written in black (W) and name different color patches (C). Conversely, in the third part, named color-word (CW) condition or incongruent condition, color-words were printed in an inconsistent color ink (i.e., the word “blue” printed in yellow) and participants were required to name the color of the ink instead of reading the word itself. The difficulty in inhibiting reading the word was called the Stroop interference effect (SIE) (35). Speed and accuracy scores were recorded for calculation of the SIE (SIE_time and SIE_accuracy) to evaluate inhibitory control ability (37). The calculation method has been detailed in (38).

Verbal fluency test (VFT) is used to measure the ability to generate words in a limited period of time, either from given semantic categories (as in the semantic VFT) or starting with certain letters (as in the phonemic VFT) (39). In this study, we gave the participants 1 min to name as many words from the category of “animals” or items starting with a certain Chinese word (Fa) as possible. The dependent measure reported was the number of words generated.

Participants were also assessed by the most reported processing speed task, the Symbol Digit Modalities Test (SDMT). The dependent measure was the number of items correctly completed within 90 s.

Statistical analyses were performed using IBM SPSS Statistics version 23.0 (Chicago, IL, USA). Age and education level were compared using two-sample *t*-tests, gender was compared using a chi-squared test, and SCWT (SIE_time and SIE_accuracy), VFT (semantic VFT and phonemic VFT), and SDMT scores between MDD patients and NCs were compared by using linear regression analyses (age, gender, and education level as covariates).

### Image Acquisition

All MRI data were acquired using a 3.0-T GE Signa HDxt scanner with an 8-channel head-coil within 3 days of diagnosis. The participants were instructed to close their eyes and refrain from thinking anything in particular. Two radiologists made consensus decisions that all participants were free of visible brain abnormalities or any form of lesions based on thick-slice axial T1- and T2-weighted images as well as T2-weighted fluid-attenuated inversion recovery (T2-FLAIR) images. In order to increase sample size, this study included two image datasets, which were acquired during two different periods but had largely the same parameters, including TR/TE = 2000/30 ms, flip angle = 90°, matrix size = 64 × 64, and slice spacing = 1.0 mm for rs-fMRI and slice thickness = 1 mm, no slice gap, matrix size = 256 × 256, field of view (FOV) = 256 × 256 mm^2^ for three-dimensional T1-weighted images (3D-T1WI). The different parameters are as follows. For the first dataset [82 MDD patients and 72 NCs, also used in (28)], the parameters included FOV = 240 × 240 mm^2^, slice thickness = 4 mm, slice number = 33, and scanning time = 8′20″ (250 volumes) for rs-fMRI and TR/TE = 10.4/4.3 ms, FA = 15°, and 156 slices for 3D-T1WI. For the second dataset (37 MDD patients and 37 NCs), the parameters included FOV = 220 × 220 mm^2^, slice thickness = 3 mm, slice number = 36, scanning time = 6′10″ (185 volumes) for rs-fMRI and TR/TE = 6.9/1.5 ms, FA = 12°, and 188 slices for 3D-T1WI. Compared to the first dataset, the second dataset has a slightly increased spatial resolution for rs-fMRI (we used the same number of volumes for each of the two datasets). The effect of different datasets was removed in the statistical analysis later.

### Image Preprocessing

Image preprocessing was performed using SPM12 (www.fil.ion.ucl.ac.uk/spm) and DPARSF version 2.3 (http://rfmri.org/DPARSF). For each rs-fMRI scan, 180 volumes remained for further analyses. The remaining images were corrected for acquisition time intervals between slices and head motion between volumes. Data from 19 MDD patients and 10 NC were discarded because their maximum cumulative head motion exceeded 2 mm in translation or 2° in rotation along any direction, or the mean framewise displacement (FD) exceeded 0.2 mm (40). Next, 3D-T1WI data were coregistered to the rs-fMRI data of the same subject and further segmented using unified segment (http://www.fil.ion.ucl.ac.uk/spm) and registered to the standard Montreal Neurological Institutes (MNI) space using diffeomorphic anatomical registration through exponentiated Lie algebra (DARTEL). The rs-fMRI data were then warped to MNI space according to the generated deformation field and smoothed with a Gaussian kernel of 6 mm full width at half maximum (FWHM). Several nuisance signals, including the Friston-24 head motion parameters and mean signals from cerebrospinal fluid and white matter, were regressed out from the rs-fMRI data. Then, linear detrending and bandpass filtering (0.01–0.08 Hz) were performed to reduce low-frequency drift and high-frequency noise.

### FC Analysis

We specified 27 predefined ROIs (see the detailed ROI definition in Table 1) from DMN, CEN, SN, and LS based on their vital role in MDD neuropathology (22, 23, 41). The coordinates of the ROIs from the DMN, CEN, and SN were adopted from Raichle (42), and those from the LS were taken from Drysdale et al. (41).

**Table 1:** Names and MNI coordinates of 27 ROIs from four networks.

**Seed**		**MNI coordinates (mm)**	**Seed**		**MNI coordinates (mm)**
**Default mode network**			**Salience network**		
1	Posterior cingulate cortex/precuneus	0 −52 7	15	Dorsal anterior cingulate cortex	0 21 36
2	Medial prefrontal cortex	−1 54 27	16	L-anterior prefrontal cortex	−35 45 30
3	L-lateral parietal cortex	−46 −66 30	17	R-anterior prefrontal cortex	32 45 30
4	R-lateral parietal cortex	49 −63 33	18	L-insula	−41 3 6
5	L-inferior temporal gyrus	−61 −24 −9	19	R-insula	41 3 6
6	R-inferior temporal gyrus	58 −24 −9	20	L-lateral parietal cortex	−62 −45 30
7	Medial dorsal thalamus	0 −12 9	21	R-lateral parietal cortex	62 −45 30
8	L-posterior cerebellum	−25 −81 −33	**Limbic system**		
9	R-posterior cerebellum	25 −81 −33	22	L-subgenual anterior cingulate cortex	−4 15 −11
**Central executive network**			23	R-subgenual anterior cingulate cortex	4 15 −11
10	Dorsal medial prefrontal cortex	0 24 46	24	L-amygdala	−19 −2 −21
11	L-anterior prefrontal cortex	−44 45 0	25	R-amygdala	19 −2 −21
12	R-anterior prefrontal cortex	44 45 0	26	L-ventral hippocampus	−27 −15 −18
13	L-superior parietal lobule	−50 −51 45	27	R-ventral hippocampus	27 −15 −18
14	R-superior parietal lobule	50 −51 45			

*R, Right; L, left*.

Using DPARSF version 2.3 (http://rfmri.org/DPARSF), we computed Pearson correlation coefficients between the mean time series of each ROI (each ROI was a sphere centering at the above coordinates with a radius of 5 mm) and that of each voxel of the whole brain. Then, a Fisher *r*-to-*z* transformation was used to convert the correlation coefficient to *z* values to improve normality. Finally, we obtained z-FC maps of each individual for further analysis. Next, we used SPM 12 (www.fil.ion.ucl.ac.uk/spm) to perform two-sample *t*-tests (gender, age, education, and center as covariates) to determine areas with significantly different FCs to the ROIs between MDD patients and NCs. We used *P* < 0.001 for the cluster-forming threshold and implemented a familywise error (FWE) correction approach at the cluster level. All results survived whole-brain cluster correction (*P*_FWE_ < 0.05).

### Correlation Between FC and Clinical Scores

The correlations between significantly different FCs and clinical scores (illness duration, HDRS-17 scores, SIE_time, SIE_accuracy, semantic VFT, phonemic VFT, and SDMT scores) were calculated using partial correlation analysis. *P* < 0.05 after Bonferroni correction was considered significant. Age, gender, and education were included as covariates in the correlation analyses of cognitive scores.

## Results

### Demographic and Clinical Characteristics

A total of 100 MDD patients (66 females, 34 males; mean age: 29.46 years) and 100 NCs (59 females, 41 males; mean age: 29.59 years) entered the following analysis, where 34 MDD patients (25 females, 9 males; mean age: 29.41 years) and 34 NCs (24 females, 10 males; mean age: 30.09 years) with neuropsychological test scores were included in the correlation analysis. No significant differences in age, gender, and education were found between the 100 MDD patients and the 100 NCs. The demographic and clinical data of 100 MDD patients and 100 NCs are summarized in Table 2.

**Table 2:** Demographic and clinical characteristics of participants.

**Characteristics**	**MDD (*N* = 100)**	**NC (*N* = 100)**	***t*/χ^2^**	***P*-value**
Age (years)	29.46 ± 9.34^§^	29.59 ± 10.33	−0.09	0.93^†^
Gender (F/M)	66/34	59/41	1.05	0.31^‡^
Education (years)	12.46 ± 3.22^§^	12.88 ± 2.77	−0.09	0.32
Illness duration (months)	8.64 ± 10.86^§^	NA	NA	NA
HDRS-17	22.15 ± 3.18^§^	NA	NA	NA

*MDD, major depressive disorder; NC, normal control; HDRS-17, 17-item hamilton depression rating scale*.

§ *Mean ± standard deviation*.

† *The P-values were obtained through a two-sample t-test*.

‡ *The P-value was obtained through a chi-squared test*.

No significant difference was found between the 34 MDD patients and the 34 NCs in terms of age, gender, education, SIE_time, SIE_accuracy or phonemic VFT scores, but the MDD patients had significantly lower semantic VFT and SDMT scores than the NCs (*P* < 0.05). See details in Table 3.

**Table 3:** Demographic and clinical characteristics of the participants with neuropsychological tests.

**Characteristics**	**MDD (*n* = 34)**	**NC (*n* = 34)**	***t*/χ^2^**	***P*-value**
Age (years)	29.41 ± 8.27^§^	30.09 ± 10.88^§^	−0.29	0.77^†^
Gender (F/M)	25/9	24/10	0.07	0.787^‡^
Education (years)	13.00 ± 3.44^§^	13.68 ± 3.07^§^	−0.86	0.395^†^
Illness duration (months)	7.81 ± 8.46^§^	NA	NA	NA
HDRS-17	21.85 ± 2.25^§^	NA	NA	NA
SIE_time	1.17 ± 0.37^§^	1.10 ± 0.32^§^	−0.95	0.35^¶^
SIE_accuracy	−0.05 ± 0.06^§^	−0.03 ± 0.04^§^	1.57	0.12^¶^
Semantic VFT	18.15 ± 5.77^§^	21.47 ± 4.82^§^	2.44	0.02^¶^
Phonemic VFT	8.15 ± 4.34^§^	9.91 ± 3.98^§^	1.86	0.07^¶^
SDMT	53.21 ± 12.28^§^	62.03 ± 14.12^§^	3.40	0.00^¶^

*MDD, major depressive disorder; NC, normal control; HDRS-17, 17-item hamilton depression rating scale; CTQ, childhood trauma questionnaire; SIE_time, interference effect of time during the Stroop test; SIE_accuracy, interference effect of accuracy during the Stroop test*.

§ *Mean ± standard deviation (SD)*.

† *The P-values were obtained by two-sample t-tests*.

‡ *The P-value was obtained by a chi-squared test*.

¶ *The P-values were obtained by linear regression analyses. Age, gender, and education level were included as covariates*.

### MDD-Related FC Alterations

**Figure 1:**
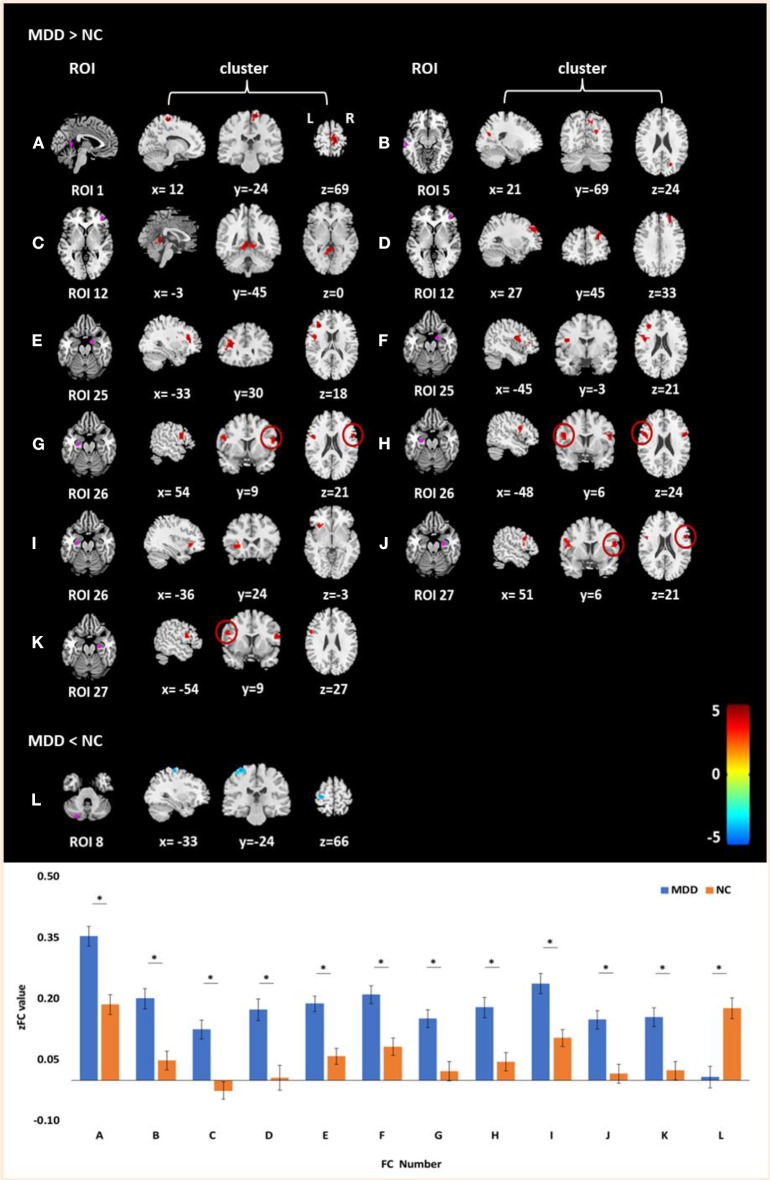
Clusters of between-group differences of FC with age, gender, education level, and center adjusted (*P* < 0.05, FWE corrected). Compared to the NCs, significantly increased FCs in MDD patients were found between **(A)** the posterior cingulate cortex/precuneus and the right paracentral gyrus; **(B)** the left inferior temporal gyrus and the right cuneus; **(C)** the right anterior prefrontal cortex and the left cerebellum_4_5 (part extend to right cerebellum_4_5); **(D)** the right anterior PFC and the right middle frontal gyrus; **(E)** the right amygdala and the left inferior frontal gyrus (triangular part); **(F)** the right amygdala and the left rolandic operculum; **(G)** the left ventral hippocampus and the right inferior frontal gyrus (opercular part); **(H)** the left ventral hippocampus and the left inferior frontal gyrus (opercular part); **(I)** the left ventral hippocampus and the left inferior frontal gyrus (orbital part); **(J)** the right ventral hippocampus and the right inferior frontal gyrus (opercular part); and **(K)** the right ventral hippocampus and the left inferior frontal gyrus (opercular part). Decreased FC in MDD patients was found between the left posterior cerebellum and the left postcentral gyrus **(L)**. Color scale denotes the *t* values; *x, y, z*, Montreal Neurological Institutes coordinates; L, left; R, right. The bar graph shows the *z* value of the above FCs (means and SD; * indicates *P* < 0.05, FWE corrected).

Significant differences were found in the FC of seven ROIs between MDD and NCs. As shown in Table 4 and Figure 1, MDD patients had higher FC than NCs between the following ROI and clusters: (A) the posterior cingulate cortex/precuneus and the right paracentral gyrus, (B) the left inferior temporal gyrus and the right cuneus, (C) the right anterior prefrontal cortex and the left cerebellum_4_5 (part extend to right cerebellum_4_5), (D) the right anterior PFC and the right middle frontal gyrus, (E) the right amygdala and the left inferior frontal gyrus (triangular part), (F) the right amygdala and the left rolandic operculum, (G) the left ventral hippocampus and the right inferior frontal gyrus (opercular part), (H) the left ventral hippocampus and the left inferior frontal gyrus (opercular part), (I) the left ventral hippocampus and the left inferior frontal gyrus (orbital part), (J) the right ventral hippocampus and the right inferior frontal gyrus (opercular part), and (K) the right ventral hippocampus and the left inferior frontal gyrus (opercular part). Additionally, lower FC was also observed between the left posterior cerebellum and the left postcentral gyrus (L).

**Table 4:** MDD-related FC alterations.

**FC number**	**Seed**	**Brain region**	**MNI coordinates (mm)**	**Cluster size**	**Peak *T***
***MDD > NC***	**Default mode network**				
A	Posterior cingulate cortex/precuneus	R-paracentral gyrus	12 −24 69	73	4.94
B	L-inferior temporal gyrus	R-cuneus	21 −69 24	72	4.06
	**Central executive network**				
C	R-anterior prefrontal gyrus	L-cerebellum_4_5	−3 −45 0	118	4.72
D		R middle frontal gyrus	27 45 33	50	4.35
	**Limbic system**				
E	R-amygdala	L-inferior frontal gyrus, triangular part	−33 30 18	90	4.80
F		L-rolandic operculum	−45 −3 21	97	4.45
G	L-ventral hippocampus	R-inferior frontal gyrus, opercular part	54 9 21	47	4.45
H		L-inferior frontal gyrus, opercular part	−48 6 24	60	4.31
I		L-inferior frontal gyrus, orbital part	−36 24 −3	44	4.08
J	R-ventral hippocampus	R-inferior frontal gyrus, opercular part	51 6 21	47	4.24
K		L-inferior frontal gyrus, opercular part	−54 9 27	59	3.80
***NC > MDD***	**Default mode network**				
L	L-posterior cerebellum	L-precentral gyrus	−33 −24 66	54	−4.35

*R, right; L, left*.

### Correlations Between Altered FC and Clinical Scores

**Figure 2:**
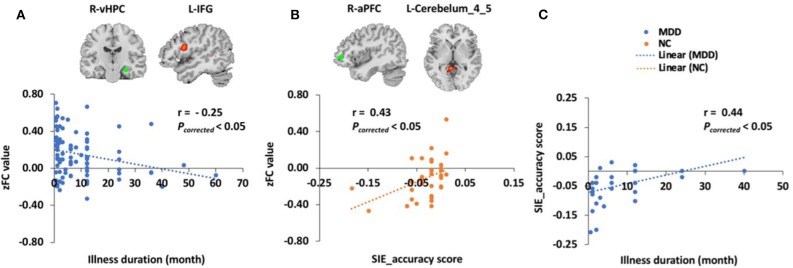
Correlations between altered FC and clinical scores. **(A)** The *z* score of the FC (zFC) between the right ventral hippocampus (R-vHPC) and the left inferior frontal gyrus (L-IFG) was negatively correlated with illness duration (*r* = −0.25, *P*_corrected_ < 0.01) in 100 MDD patients. **(B)** The zFC between the right anterior prefrontal cortex (R-aPFC) and the left cerebellum_4_5 (L-cerebellum_4_5) was positively correlated with the SIE_accuracy score (*r* = 0.43, *P*_corrected_ < 0.05) in the 34 NCs. **(C)** The SIE_accuracy score was correlated with illness duration in the 34 MDD patients (*r* = 0.44, *P*_corrected_ < 0.05).

For all 100 MDD patients, FC between the right ventral hippocampus in the LS and the left inferior frontal gyrus in the CEN was negatively correlated with illness duration (*r* = −0.25, *P*_corrected_ = 0.01). In the 34 MDD patients and 34 NCs with neuropsychological test scores, the SIE_accuracy score was correlated with illness duration in the MDD group (*r* = 0.44, *P*_corrected_ = 0.03). Although there was no difference in SIE_time or SIE_accuracy scores between the two groups, we still found that the SIE_accuracy score was positively correlated with the FC between the right anterior prefrontal cortex in the CEN and the left cerebellum_4_5 in the visual network (*r* = 0.43, *P*_corrected_ = 0.03) in the NCs but not the MDD patients (Figure 2). In addition, there were significant differences in semantic VFT and SCWT scores between the two groups. However, no correlation was found between FCs and HRDS-17, semantic VFT, phonemic VFT, or SDMT scores.

## Discussion

In this study, we analyzed the FC differences of 27 seeds from the DMN, CEN, SN, and LS with the voxels of the whole brain between 100 first-episode, drug-naïve MDD patients and 100 NCs. We also correlated the significantly altered FC with scores on a battery of neuropsychological tests that covered cognitive domains including executive function, verbal fluency, and processing speed in 34 MDD patients and 34 NCs. The result showed that significant FC differences between groups were identified among the seeds and clusters in the DMN, CEN, LS, visual network, somatomotor network, ventral attention network, and dorsal attention network. In the MDD patients, the magnitude of the Stroop interference effect was positively correlated with the illness duration, and the illness duration was negatively correlated with the FC between the right ventral hippocampal gyrus and the left inferior frontal gyrus. However, the correlation between the Stroop interference effect and the FC of the right anterior prefrontal cortex with the left cerebellum_4_5 was disrupted in the MDD patients. Our findings offer a novel insight into the pathophysiological mechanisms of executive function in MDD.

### MDD-Related FC Alterations

The DMN, CEN, SN, and LS support emotion regulation and higher cognitive functions in MDD (43). In this study, we observed several discriminative brain regions contributing to MDD-related FC alterations, including the posterior cingulate cortex, left inferior temporal gyrus and left posterior cerebellum in the DMN, the right anterior prefrontal cortex in the CEN, and the right amygdala and bilateral ventral hippocampus in the LS. The DMN provides the neural substrate for depressive rumination and is the network that receives the most attention in clinical MDD imaging research (44, 45). In our study, the results showed that MDD patients had altered FC between the DMN and the somatomotor network—we reported the novel finding of increased FC between the posterior cingulate cortex and the right paracentral gyrus, while decreased FC between the left posterior cerebellum and left precentral gyrus in MDD patients. The posterior cingulate cortex plays a pivotal role in the DMN (46), and from the current body of research, it has been demonstrated to have increased engagement in MDD and predicts disease severity (47, 48). The left posterior cerebellum (crus II) is believed to be coupled with the DMN, showing a possible role in memory and planning processing (49). Also, according to our previous study, crus II may be a promising biomarker for MDD diagnosis (28). The paracentral gyrus and precentral gyrus belong to the somatomotor network. They are not simply motor structures but also involved in more “cognitive” processes, including response inhibition, action sequencing, working memory, speech and language processing (50–52). Previous studies have reported the altered FC between the DMN and somatomotor network. Bessette et al. (53) found that the remitted MDD patients had weaker connectivity between the DMN seed (right hippocampus) and the SMN seed (right paracentral lobule). These altered FCs between the DMN and SMN may reflect ongoing rumination and underlie deficits in cognitive control. Besides, in MDD patients, altered FC was also found between the DMN and the visual network. Our result showed an increased FC between the left inferior temporal gyrus and the right cuneus, suggesting abnormal processing in the DMN and visual network in MDD. This was contradicting to the results of other studies; they found reduced FC between these two networks in MDD patients (45). However, most of these results were contributed by recurrent MDD patients, not by first-episode and drug-naïve patients, which need to be confirmed by more future studies.

Multiple MDD studies have focused on other typically impaired brain networks such as CEN and LS because of their roles in emotion processing, executive functioning and antidepressant action (21). Our results also indicated that the fronto-limbic system has altered in the first-episode and drug-naïve MDD patients. We found increased FC of the right amygdala with the left inferior frontal gyrus and the left rolandic operculum, and increased FC of bilateral ventral hippocampus with the bilateral inferior frontal gyrus. The amygdala and hippocampus are the core regions in the LS and have widespread connections to diverse cortical areas, such as the frontal cortex, which is the region known to constitute the neuroanatomical network of cognitive function (54). In addition, increased FC of CEN with ventral attention network and visual network, and increased FC of LS with dorsal attention network may reflect altered or biased salience monitoring.

### Relationship Between Altered FC and Clinical Scores

SCWT is a famous test for measuring the executive function, especially inhibition (55). In the SCWT, the difficulty in inhibiting reading the word in the incongruent condition was called the SIE (35). Traditionally, MDD patients had higher SIE score than the NCs, indicating poor executive function in MDD (36). An event-related fMRI study concluded that higher SIE was correlated with reduced activation in the dorsal anterior cingulate cortex and the left dorsolateral prefrontal cortex (56). In our study, no difference of SIE was found between the MDD patients and the NCs, which is in accordance with the result of Wagner et al. (57), but the correlation between the SIE and the FC of the right anterior prefrontal cortex with the left cerebellum_4_5 (part extend to the right cerebellum_4_5) was disrupted in the patients. The disrupted correlation indicates that the altered FC may present before symptoms develop, suggesting that it is the core process in the development of executive function impairment rather than being produced by the symptoms. In addition, we found that in the MDD patients, the SIE accuracy was related to the illness duration. However, the inconsistency of the above studies highlighted the importance of replicating the results of previous studies (58). Besides, we found that the longer illness duration in MDD was correlated with decreased FC between the right ventral hippocampus and the left inferior frontal gyrus in the MDD patients, supporting the notion that the fronto-limbic system is the key network in MDD (21). Collectively, our observation may indicate that altered FC of seeds in the CEN and LS could be associated with illness duration and executive function impairment via wide-ranging connections to cortical and subcortical brain regions.

In our study, MDD patients produced significantly fewer words than NCs in the semantic VFT but not in the phonemic VFT. Some previous studies indicated that MDD patients had worse performance than NCs in both the semantic and phonemic VFT, but with a significantly larger effect size for semantic fluency (59–61). Our study also showed that the SDMT score was significantly lower in MDD patients than in NCs, indicating a slower processing speed of MDD patients (62–64). We may need other metrics from fMRI to study the above cognitive impairments since no correlation was found between the altered FCs and VFT or SDMT scores in the present study.

### Limitations

Our study had some limitations. First, we did not compute the sample size formula before the experiment. Although the sample size of MDD patients in our study was larger than those of most MDD studies, it is still insufficient, especially the number of MDD patients with available cognitive domain scores. The very small sample size will reduce the statistical power and the reproducibility of a study (65). We will do sample size calculation and continue to recruit more MDD patients with cognitive scores in further work. Second, we did not divide the MDD patients into mild, moderate, and severe depression subgroups. As we know, more subgroups of MDD subjects based on the disease severity could lead to more meaningful findings, as the depression severity may also be correlated to a different degree of cognitive impairments. However, in our study, only moderate and severe subgroups could be identified from the patient group. Besides, creating different subgroups will cause small sample size in each subgroup, which could negatively affect the result of our study. We will recruit mild depression patients in further work and separately investigate different subgroups. Besides, we only recruited only first-episode, drug-naïve MDD patients. Selecting this group of MDD patients eliminates possible confounding factors such as illness duration and medication use (66). However, different MDD subtypes could have different neurobiological mechanisms and should be investigated separately in the future (67). Third, we used only one imaging modality, but other modalities also provide valuable diagnostic information and could be used jointly with our protocol in order to improve diagnosis. In addition, conventional FC is commonly used in fMRI studies, but if we wish to further our understanding of its biological meaning, other advanced methods, such as dynamic FC and high-order FC, should be applied to our future MDD study.

## Conclusions

The MDD patients have altered FCs among multiple brain networks and a disrupted correlation between the FC of prefrontal cortex and executive function. The disrupted correlation could present before the symptoms develop and may be the core process in the development of executive function impairment. This study offers a novel insight into the pathophysiological mechanisms of executive function impairment in MDD.

## Data Availability Statement

The datasets generated for this study are available on request to the corresponding author.

## Ethics Statement

The studies involving human participants were reviewed and approved by The First Affiliated Hospital of Guangzhou University of Chinese Medicine. The patients/participants provided their written informed consent to participate in this study.

## Author Contributions

YL, YCh, XL, and SQ contributed to conception and design of the study. DL, YZ, HZ, YCu, JC, and JL organized the data. YL performed the data analysis and drafted the manuscript. All authors revised the manuscript, and read and approved the submitted version.

## Conflict of Interest

The authors declare that the research was conducted in the absence of any commercial or financial relationships that could be construed as a potential conflict of interest.
